# The Treatment of Hemorrhagic Disease of the New-Born by Direct Transfusion of Blood

**Published:** 1914-09

**Authors:** 


**The Treatment of Hemorrhagic Disease of the New-born by Direct Transfusion of Blood.** Lespinasse, in the J. of the A.

M. A., June 13, 1914, reports fifteen cases with no death due to the transfusion, two babies died however a few days later from congenital lues. In the donor the bloodvessels used were the radial artery in seven cases and a forearm vein in seven cases; on the baby the femoral vein was used in four and the jugular in ten; the duration of the transfusion was from five to fifteen minutes.—C. G. L.-W.

